# Influenza among U.K. Pilgrims to Hajj, 2003

**DOI:** 10.3201/eid1010.040151

**Published:** 2004-10

**Authors:** Haitham El Bashir, Elizabeth Haworth, Maria Zambon, Shuja Shafi, Jane Zuckerman, Robert Booy

**Affiliations:** * Queen Mary’s School of Medicine and Dentistry at Barts and The London, London, United Kingdom;; † Health Protection Agency, London, United Kingdom;; ‡ Royal Free and University College Medical School, London, United Kingdom

**Keywords:** letter, hajj, influenza, outbreak, pilgrim, Saudi Arabia

**To the Editor:** Each year, approximately 2 million Muslims travel from all over the world to participate in hajj. Approximately 22,000 pilgrims travel from the United Kingdom to Makkah, Saudi Arabia; of those, approximately 1,000 pilgrims reside in the east end of London. In the past, infectious diseases research conducted during these pilgrimages focused on meningococcal disease because of outbreaks associated with the hajj. Since 2000, the dates of the hajj have been moved back into the winter season; this time change could lead to a seasonal increase in outbreaks of respiratory infections caused by influenza and other viruses. From 1991 to 1992, influenza A was a common cause of respiratory infection in pilgrims tested in Makkah (*1*). However, the incidence rate of influenza among pilgrims from Europe is not well-known. A previous study of influenzalike illness among pilgrims from Pakistan reported rates of 36% in influenza-vaccinated pilgrims and 62% in influenza-nonvaccinated pilgrims; these results were based on clinical endpoints without microbiologic confirmation (*2*).

We assessed the risk for influenza infection among a cohort of pilgrims from the east end of London who participated in the hajj in 2003. From December 2002 to January 2003, we enrolled 115 participants who planned to take part in hajj in 2003. The study was approved by the North London Multicentre Research Ethics Committee and the Trustees of East London Mosque. Informed consent was obtained through appropriate translators. All participants attended the East London Mosque, Whitechapel, London; 30 were vaccinated with influenza vaccine (A/New Caledonia/20/99 [H1N1]-like strain, A /Moscow/10/99 [H3N2]-like strain, B/Sichuan/379/99-like strain). Venous blood samples were collected, and questionnaires were completed before the participants departed for the hajj and within 2–3 weeks of their return in February to March 2003.

Tests for influenza A and B were conducted by using hemagglutination inhibition against the following influenza antigens: A/NewCalidonia/20/99, A/Wuhan/371/91, A/Sydney/5/97, A/Panama/2007/99, B/Sichuan/379/99, and B/Harbin/7/94. A diagnosis of influenza was made based on seroconversion with at least a fourfold rise in antibody titer. Based on seroconversion, the influenza attack rate among all pilgrims was 38% (44/115). The attack rate was 30% among the vaccinated and 41% among the nonvaccinated participants (Table) (odds ratio for influenza in vaccinees = 0.61, p = 0.28). Of the 44 patients, 42 (37%) were infected with influenza A H3N2; 1 had influenza A H1N1, and 1 had influenza B infection. Six influenza A H3N2 patients were dually infected; two patients seroconverted to A H1N1, and four patients seroconverted to influenza B. Nearly half (21/44) of the patients with influenza received a course of antimicrobial drugs while on the hajj compared with 38% (27/71) of those who did not seroconvert. The attack rate in the vaccinated patients was lower than the rate in nonvaccinated patients, which is consistent with some protective effect of the influenza vaccine.

**Table:** Seroconversion and respiratory symptoms due to influenza infection and vaccination status among U.K. pilgrims

Influenza vaccination in autumn 2002	Seroconversion		Respiratory symptoms	
	Yes	No	Yes	No
Vaccinated	9	21	23	7
Nonvaccinated	35	50	70	15
Total	44	71	93	22

Even though blood was collected from five convalescing patients within 3 weeks of their return from the hajj, some of the patients may have acquired influenza B infection immediately after their return to the United Kingdom, as it was the main strain circulating in the United Kingdom in late February to March 2003. Many pilgrims from throughout the world, some of whom may carry H3N2 drift variants, mingle closely during the hajj. This type of exposure increases the risk for worldwide spread of new drift variants and other contagious respiratory diseases (*3*). Given the potential for the high influenza attack rate documented in this study, all pilgrims, regardless of age, should be offered influenza vaccination before they travel on the hajj during winter months. On-site testing for influenza should be available to medical services in Makkah (and countries of origin), and treatment with a neuraminidase inhibitor should be offered to persons who test positive and have been symptomatic for <48 hours (*4*). This treatment should lessen the transmission risk to pilgrims during the crowded events during travel and on their return home (*5*). When pilgrims return from the hajj, physicians should be informed that pilgrims may bring back new drift variants of influenza; physicians should consider the diagnosis and treat persons at risk and their close contacts (*4*).
